# Inflammatory and Nested Testicular Sex Cord Tumors: Clinical and Molecular Characterization

**DOI:** 10.3390/genes17030340

**Published:** 2026-03-19

**Authors:** Panagiotis J. Vlachostergios, Foteini Karasavvidou, Konstantinos Evmorfopoulos, Ioannis Zachos, Vassilios Tzortzis

**Affiliations:** 1 Department of Medical Oncology, IASO Thessalias Hospital, 41500 Larissa, Greece; 2 Department of Urology, Faculty of Medicine, University of Thessaly, 41110 Larissa, Greece; 3 Department of Medicine, Division of Hematology and Medical Oncology, Weill Cornell Medicine, New York, NY 10065, USA; 4 Department of Pathology, University Hospital of Larissa, 41110 Larissa, Greece

**Keywords:** inflammatory and nested testicular sex cord tumor, testicular sex cord–stromal tumor, EWSR1::ATF1 fusion, testicular neoplasm, molecular pathology

## Abstract

Inflammatory and nested testicular sex cord tumor (IN-TSCT) is a recently characterized malignant neoplasm within the spectrum of testicular sex cord–stromal tumors. Previously misclassified as Sertoli cell tumor, not otherwise specified, or as seminoma, this entity has emerged as a distinct clinicopathologic and molecular subtype defined by recurrent *EWSR1::ATF1* gene fusions and a potentially aggressive clinical course. Patients most commonly present with unilateral painless testicular enlargement, and radiologic findings are typically nonspecific. Histologically, tumors demonstrate solid and nested growth patterns, epithelioid cytology with eosinophilic to clear cytoplasm, prominent hyalinized stroma, and a conspicuous inflammatory infiltrate. Immunophenotypically, tumors express sex cord–stromal markers, including steroidogenic factor-1 (SF-1) and inhibin, and frequently co-express epithelial membrane antigen and CD30 while lacking germ cell tumor markers. Molecular studies indicate fusion-driven oncogenesis associated with low tumor mutational burden. Published cases suggest that IN-TSCT may exhibit aggressive clinical behavior, including metastatic spread in a subset of patients; however, the total number of reported cases remains very limited, and the true metastatic risk and prognostic spectrum have not yet been clearly defined. This review synthesizes the available literature to provide a comprehensive clinicopathologic and molecular overview of this emerging tumor entity.

## 1. Introduction

Testicular sex cord–stromal tumors (SCSTs) comprise a rare and heterogeneous group of neoplasms derived from the supporting cells of the testis, including Sertoli, Leydig, and granulosa cell lineages [1,2,3]. Historically, their classification has relied largely on morphologic features; however, advances in molecular pathology have revealed genetically distinct subsets within morphologically overlapping categories. Recent genomic studies have identified recurrent alterations such as *CTNNB1* mutations in Sertoli cell tumors and *PRKAR1A* alterations in large cell calcifying Sertoli cell tumors, underscoring the importance of an integrated morphologic and molecular classification [1,2,3].

Inflammatory and nested testicular sex cord tumor (IN-TSCT) is a recently described malignant subtype of testicular sex cord–stromal tumor (TSCT) characterized by distinctive histopathologic and molecular features. These tumors typically form solid nests and sheets of epithelioid cells with granular eosinophilic to clear or vacuolated cytoplasm and are commonly accompanied by a prominent inflammatory infiltrate composed of lymphocytes, plasma cells, eosinophils, and neutrophils. The stroma is often collagenous and may show hyalinization. Tumor necrosis and local invasion are frequently observed, whereas mitotic activity is generally low [1].

Immunophenotypically, these tumors are characteristically positive for α-inhibin, steroidogenic factor-1 (SF-1), epithelial membrane antigen, and often CD30, but lack nuclear β-catenin expression. Molecularly, they are defined by recurrent *EWSR1::ATF1* gene fusions or *ATF1* amplification, distinguishing them from classic Sertoli cell tumors and other TSCTs. Clinically, inflammatory and nested TSCTs appear to exhibit aggressive behavior in a subset of reported cases, with metastases involving retroperitoneal and nonretroperitoneal lymph nodes, bone, the contralateral testis, and lung [1].

This entity is not yet formally included in the current World Health Organization (WHO) classification, but recent expert analyses and molecular studies support its recognition as a distinct diagnostic category because of its unique morphologic, immunophenotypic, and clinical characteristics.

In this review, we integrate clinical, histopathologic, and genomic data from the 15 cases reported to date worldwide together with a recent case from our institution (UTh), for which comprehensive next-generation sequencing (NGS), germline testing, and immunohistochemical (IHC) profiling were performed. Recognition of this entity is clinically important because it is frequently misdiagnosed as seminoma or lymphoma, which may lead to inappropriate or suboptimal management.

## 2. Clinical Presentation and Natural History

Patients with IN-TSCT span a broad adult age range, with reported cases occurring from the second to the eighth decades of life and a median age in the mid-30s [1]. The most common clinical presentation is unilateral painless testicular swelling or a palpable mass. Constitutional symptoms are typically absent. Serum germ cell tumor markers are usually within normal limits, reflecting the non-germ cell origin of the tumor [1].

Radiologic evaluation generally demonstrates a solid intratesticular mass without specific distinguishing imaging features. Because imaging lacks sufficient discriminatory value, histopathologic examination remains the cornerstone of diagnosis. In several early reports, a significant proportion of tumors were initially misdiagnosed as seminoma, highlighting the clinical impact of morphologic overlap and diagnostic mimicry [1,2,3,4].

These tumors may demonstrate local invasion and the potential for metastatic spread. Metastases can be present at diagnosis or may develop early during the disease course, and several reported patients have developed metastatic disease during follow-up. The retroperitoneal lymph nodes represent the most common site of metastasis, followed by extra-retroperitoneal nodal stations, lung, bone, and occasionally the contralateral testis [1,4,5,6]. Compared with other testicular sex cord–stromal tumors (TSCTs), inflammatory and nested tumors appear to exhibit more aggressive clinical behavior in a subset of reported cases, potentially associated with less favorable outcomes [1,6].

When IN-TSCT is confined to the testis, orchiectomy with or without adjuvant platinum-based chemotherapy has been reported as an effective therapeutic approach [1,7,8]. However, disease recurrence and metastatic progression have been associated with poor outcomes in some reported cases [1,7,9,10]. Nevertheless, because the total number of reported cases remains very small, currently available data are insufficient to accurately define the true metastatic risk and prognostic spectrum of this entity. Although published cases suggest that IN-TSCT may exhibit aggressive clinical behavior, including metastatic spread in a subset of patients, the overall natural history of the disease remains incompletely characterized.

## 3. Pathology

Gross examination typically reveals a solid, well-circumscribed intratesticular mass. Cut surfaces are variably tan-white to yellow and often show firm fibrotic or hyalinized areas. Foci of necrosis may be identified, whereas hemorrhage is uncommon but has been reported. No gross features are pathognomonic, and a definitive diagnosis requires histopathologic and molecular evaluation [2,3].

### 3.1. Histopathologic Features

**Figure 1:**
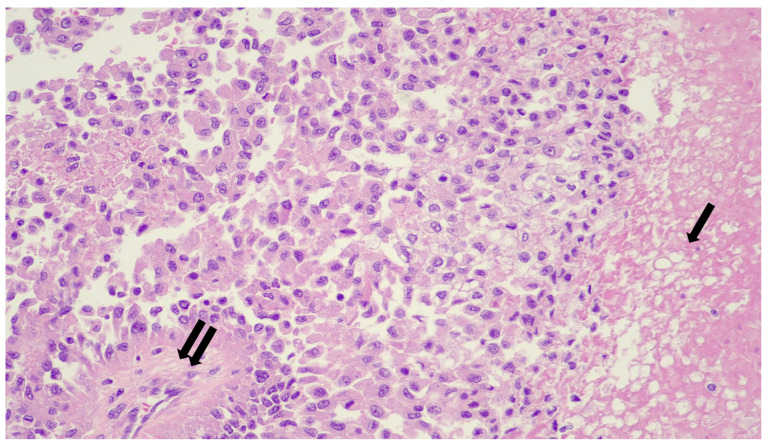
UTh patient: Cellular neoplasm composed of epithelioid cells predominantly arranged in sheets and nests. Focally, a group of cells imparting a pseudopapillary architecture (double arrow). The tumor cells show clear-to-eosinophilic cytoplasm and have rounded vesicular nuclei with variably prominent nucleoli. There are scattered admixed lymphocytes. Area of necrosis is also present (arrow). (H/E X200.)

Microscopically, IN-TSCT exhibits a combination of solid, nested, trabecular, and sheet-like architectural patterns. Tumor nests may be lobulated and are frequently separated by dense collagenous or hyalinized stroma (Figure 1). Focal discohesion within tumor nests can create pseudoglandular or pseudotubular spaces. An intratubular growth component has been described in a subset of cases, further supporting sex cord–stromal differentiation [1,7].

Tumor cells are predominantly epithelioid and display abundant eosinophilic to clear or vacuolated cytoplasm. High-power examination often demonstrates delicate cytoplasmic clearing that imparts a characteristic “spider-web” appearance. Intracytoplasmic hyaline inclusions are frequently observed but are not present in all cases. Nuclear atypia is generally mild to moderate, with smaller nuclei and less conspicuous nucleoli compared with those seen in seminoma. Mitotic activity is typically low, despite the clinically malignant behavior of the tumor [1,7].

The stroma represents a defining morphologic feature, often demonstrating dense hyalinization and stromal sclerosis. A prominent inflammatory infiltrate is consistently present and typically includes lymphocytes, plasma cells, eosinophils, and neutrophils. Occasional granulomas or aggregates of foamy histiocytes have also been described. Tumor necrosis is common and may be extensive in advanced cases [1,7].

### 3.2. Immunophenotype

Formalin-fixed, paraffin-embedded (FFPE) tissue sections from reported cases of IN-TSCT (*n* = 16) were analyzed using immunohistochemistry (IHC) according to standardized protocols, including antigen retrieval, blocking, and incubation with primary antibodies [1,8,9,10]. The antibody panel included SF-1, vimentin, inhibin, EMA, CD30, OCT3/4, SALL4, WT1, PD-L1, HER2, and ALK, with results interpreted by experienced pathologists.

Immunohistochemical studies support sex cord–stromal differentiation while also highlighting diagnostically useful aberrant expression patterns. Vimentin immunohistochemistry serves as a sensitive marker of mesenchymal and sex cord–stromal differentiation, and its expression supports the diagnosis of testicular sex cord–stromal tumors, including inflammatory and nested variants. Vimentin is consistently expressed in normal Sertoli cells, Leydig cells, and most sex cord–stromal tumors, reflecting their mesenchymal origin and differentiation profile [11,12,13].

**Figure 2:**
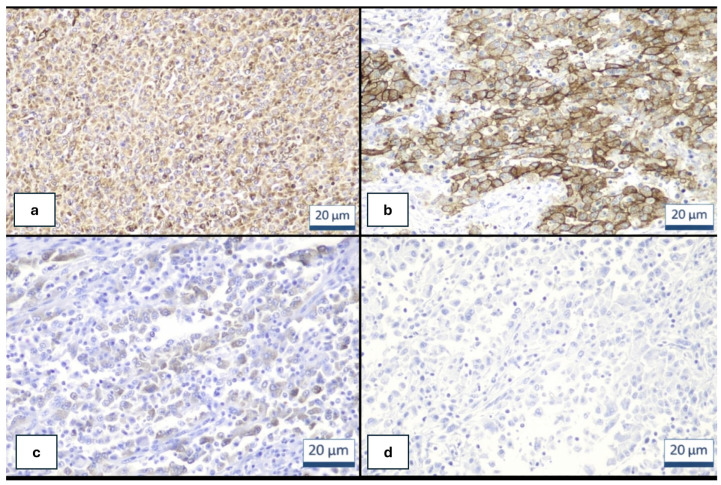
UTh patient: (**a**) Cytoplasmic immunohistochemical staining for vimentin (Vimentin X100). (**b**) Strong and diffuse membranous immunohistochemical staining for CD30 (CD30X200). (**c**) Scattered immunohistochemical staining for inhibin (Inhibin X100). (**d**) Tumor cells were negative for SALLA4 (SALLA4 X100).

In the context of inflammatory and nested testicular sex cord tumors, vimentin positivity may assist in distinguishing these neoplasms from epithelial tumors and germ cell tumors, which typically demonstrate absent or only focal vimentin expression [13,14,15]. However, vimentin is not specific for sex cord–stromal tumors, as it is also expressed in a broad range of mesenchymal neoplasms and certain carcinomas. Therefore, the diagnostic value of vimentin lies in its use as part of a broader immunohistochemical panel—including α-inhibin, steroidogenic factor-1 (SF-1), and CD30—to confirm sex cord–stromal lineage and to distinguish inflammatory and nested testicular sex cord tumors from histologic mimics [1,11,12]. Vimentin alone cannot reliably subclassify sex cord–stromal tumors or differentiate inflammatory and nested variants from other subtypes, but its positivity provides supportive evidence in the diagnostic workup (Figure 2a).

CD30 expression is present in the majority of reported cases and may show diffuse staining (Figure 2b). This finding represents an important diagnostic pitfall, as it may lead to misclassification as anaplastic large cell lymphoma. Tumor cells typically demonstrate diffuse nuclear expression of steroidogenic factor-1 (SF-1) and frequent positivity for WT1 and α-inhibin (Figure 2c). Diffuse epithelial membrane antigen (EMA) expression is also characteristic, an unusual feature among sex cord–stromal tumors that may create diagnostic confusion with carcinoma [1,8,9,10].

Cytokeratin expression is variable, whereas nuclear accumulation of β-catenin is absent, helping distinguish IN-TSCT from *CTNNB1*-mutant Sertoli cell tumors. Germ cell tumor markers—including OCT3/4, placental alkaline phosphatase (PLAP), and SALL4—are consistently negative (Figure 2d). This immunophenotypic profile is critical for differentiating IN-TSCT from seminoma and other germ cell tumors.

**Figure 3:**
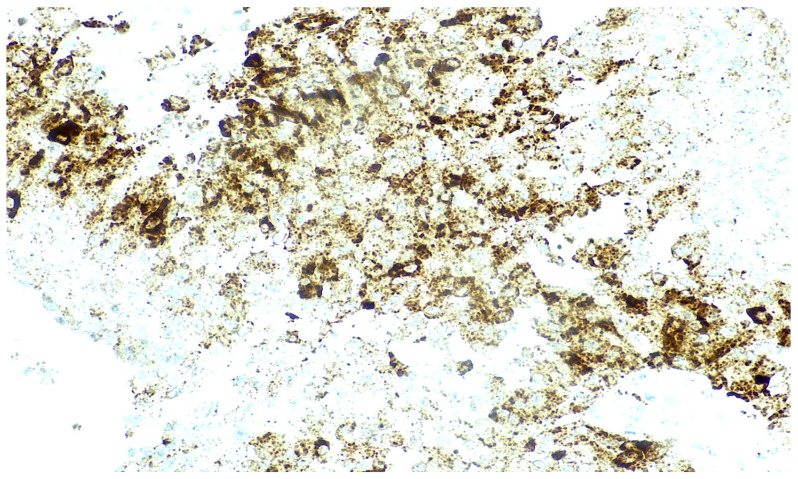
UTh patient: Intensely positive granular cytoplasmic ALK staining in a large proportion of neoplastic cells (ALK X200).

Anaplastic lymphoma kinase (ALK) expression in IN-TSCT represents an emerging and incompletely characterized immunophenotypic feature. Strong diffuse cytoplasmic ALK expression by immunohistochemistry has been reported in 1 of 16 cases, in the absence of detectable *ALK* gene rearrangement [10] (Figure 3). This pattern suggests protein overexpression rather than canonical ALK fusion-driven oncogenesis.

The therapeutic relevance of ALK expression in IN-TSCT remains uncertain. In tumors such as *ALK*-rearranged non-small cell lung carcinoma and anaplastic large cell lymphoma (ALCL), ALK inhibitors have demonstrated substantial clinical efficacy. However, in the absence of confirmed ALK genomic activation, there is currently no evidence supporting the use of targeted ALK inhibition in IN-TSCT.

Likewise, although strong diffuse cytoplasmic ALK expression has been reported in isolated IN-TSCT cases, genomic analyses have not identified ALK rearrangements or activating mutations. Therefore, ALK immunoreactivity alone should not be interpreted as evidence of a therapeutically targetable oncogenic driver, and ALK inhibitors currently have no established role in the management of IN-TSCT without confirmed genomic alterations.

## 4. Molecular Genetics

The recognition of IN-TSCT as a molecularly defined entity highlights the critical importance of tumor next-generation sequencing (NGS) in diagnostically challenging testicular neoplasms. While histomorphology and immunohistochemistry remain the foundation of diagnosis, IN-TSCT is fundamentally a fusion-driven tumor characterized by recurrent *EWSR1::ATF1* gene rearrangements. Consequently, molecular confirmation is not merely adjunctive but often decisive for establishing the diagnosis. Comprehensive genomic profiling may also identify potentially actionable co-alterations in rare cases.

Among the reported cases in the literature, tumor NGS has been performed using several validated targeted sequencing platforms, including a 282-gene panel (OncoPanel) in three cases, a 523-gene panel (HopeSeq) in one case, and a 1021-gene panel with 38 fusion targets (PrimeDx) in the most recent case from our institution. The defining molecular hallmark of IN-TSCT is rearrangement of *EWSR1*, most commonly resulting in an *EWSR1::ATF1* fusion. This in-frame fusion juxtaposes the transcriptional activation domain of EWSR1 with the DNA-binding domain of ATF1, resulting in aberrant transcriptional activation of downstream target genes. Fusion detection has been achieved using fluorescence in situ hybridization (FISH) and next-generation sequencing-based platforms.

Genomic profiling generally reveals a relatively quiet mutational landscape, characterized by low tumor mutational burden and microsatellite stability. Recurrent secondary mutations appear to be uncommon. Isolated reports have described pathogenic *TP53* alterations and germline *CHEK2* variants, although their biological and clinical significance remains uncertain (Table 1). Rare fusion-negative tumors demonstrating *ATF1* amplification have also been described, suggesting alternative mechanisms that activate the same transcriptional pathway. Of note, SF-1 negativity has been reported in 1 of the 16 cases described to date, which is notable because most previously reported IN-TSCTs demonstrate SF-1 positivity. This observation highlights phenotypic variability and further reinforces the importance of molecular confirmation for accurate diagnosis.

Collectively, molecular findings indicate that IN-TSCT is a fusion-driven tumor with a relatively genomically quiet profile, although occasional alterations affecting homologous recombination DNA repair pathways may provide potential opportunities for therapeutic targeting.

## 5. Differential Diagnosis

The principal histologic differential diagnosis is seminoma, given the shared nested architecture and associated lymphoid infiltrates. However, seminoma typically exhibits fibrous septa, a relatively uniform lymphocytic inflammatory infiltrate, prominent nucleoli, and strong expression of germ cell tumor markers. In contrast, IN-TSCT demonstrates a mixed inflammatory infiltrate, hyalinized stroma, and positivity for sex cord–stromal markers.

Sertoli cell tumor, not otherwise specified, also represents an important differential diagnostic consideration. Unlike IN-TSCT, many Sertoli cell tumors harbor *CTNNB1* mutations with nuclear β-catenin accumulation and lack *EWSR1* rearrangements.

CD30 positivity in IN-TSCT may raise the consideration of lymphoma, particularly anaplastic large cell lymphoma (ALCL). However, demonstration of steroidogenic factor-1 (SF-1) and inhibin expression, together with the absence of leukocyte markers, readily resolves this diagnostic distinction. Additional considerations include metastatic carcinoma and paratesticular mesothelioma, although clinicopathologic correlation and molecular testing are typically decisive in establishing the correct diagnosis [1,2,3,7].

## 6. Comparative Genomics and Cross-Gonadal Parallels in Sex Cord–Stromal Tumors

Sex cord–stromal tumors of the testis and ovary share a common embryologic origin, arising from gonadal supporting cell lineages. Consequently, tumors arising in these organs often exhibit overlapping morphologic features, immunophenotypic profiles (Table 2), and, increasingly, shared molecular alterations.

Comparative genomic analysis has become an essential tool for refining tumor classification, clarifying lineage relationships, and identifying therapeutically relevant pathways [16]. Among ovarian SCSTs, adult granulosa cell tumor is the best-characterized entity at the molecular level. More than 95% of cases harbor the pathognomonic *FOXL2* c.402C>G (p.C134W) mutation, which drives tumorigenesis through transcriptional dysregulation and altered TGF-β signaling pathways. This mutation is highly specific and is widely considered a defining diagnostic marker [17,18]. Juvenile granulosa cell tumors, in contrast, lack *FOXL2* mutations and instead demonstrate alterations involving *AKT1*, *GNAS*, or *DICER1*, highlighting age-dependent molecular divergence within granulosa cell tumors [19,20].

Sertoli–Leydig cell tumors of the ovary provide another informative molecular parallel. A substantial subset harbors *DICER1* mutations, frequently of germline origin, linking these neoplasms to the DICER1 tumor predisposition syndrome [21]. These tumors demonstrate aberrant microRNA processing and dysregulated cellular differentiation, reinforcing the importance of post-transcriptional regulatory mechanisms in sex cord–stromal tumorigenesis [22].

Testicular SCSTs share some overlapping molecular pathways but also exhibit organ-specific oncogenic drivers. For example, *CTNNB1* mutations characterize a subset of Sertoli cell tumors, leading to nuclear accumulation of β-catenin and activation of the Wnt signaling pathway [23,24]. Large cell calcifying Sertoli cell tumors frequently harbor *PRKAR1A* alterations, particularly in patients with Carney complex [25].

Within this comparative framework, inflammatory and nested testicular sex cord tumor (IN-TSCT) appears molecularly distinct. Rather than mutation-driven oncogenesis typical of many ovarian SCSTs, IN-TSCT is defined by recurrent *EWSR1::ATF1* gene fusions [1]. This places IN-TSCT within the broader family of *CREB*-fusion-driven neoplasms, rather than within the classical gonadal stromal tumor pathways. The transcriptional consequences of *EWSR1::ATF1* fusions likely converge on cAMP-responsive and cellular stress-response signaling pathways, representing a mechanistic axis not previously central to gonadal SCST biology. Key molecular drivers across testicular and ovarian SCSTs are summarized in Table 3.

This divergence suggests that IN-TSCT may represent a fusion-defined lineage within the sex cord–stromal tumor (SCST) spectrum, analogous to other fusion-driven neoplasms such as sarcomas, and underscores the importance of molecular testing in diagnostically challenging cases.

Comparative analysis of gonadal sex cord–stromal tumors highlights both shared lineage biology and significant molecular divergence. While many ovarian SCSTs are driven by recurrent point mutations affecting transcriptional or microRNA regulatory networks, inflammatory and nested testicular sex cord tumor is characterized by a fusion-driven oncogenic mechanism centered on the *EWSR1::ATF1* gene fusion. This distinction not only supports its classification as a unique molecular entity but also broadens the genomic landscape of gonadal stromal neoplasia.

Future studies integrating transcriptomic profiling and epigenetic mapping across gonadal tumor types may clarify whether CREB-family fusion-driven tumors represent a previously unrecognized developmental branch within sex cord–stromal tumorigenesis.

## 7. Management Strategies

The cornerstone of management is primary surgical excision via radical orchiectomy [4,5,6,26]. Given the potentially aggressive clinical behavior and risk of metastatic spread, early retroperitoneal lymph node dissection (RPLND) may be considered in patients with high-risk features or clinical evidence of nodal involvement [4,5,6,26]. The role of RPLND remains controversial; however, early intervention in patients with documented nodal metastases may improve clinical outcomes [4,5]. Although published reports suggest that IN-TSCT may demonstrate aggressive behavior, including metastatic spread in a subset of patients, the overall number of reported cases remains very small, and the true metastatic risk and prognostic spectrum are not yet well defined.

Systemic therapy outcomes are also poorly characterized. Malignant SCSTs, including IN-TSCT, appear relatively resistant to conventional germ cell tumor chemotherapy regimens. Platinum-based chemotherapy has been used in reported cases, with variable and often limited clinical benefit. CD30 expression raises the theoretical possibility of targeted therapy with brentuximab vedotin, although current evidence supporting this approach remains anecdotal. Overall, chemotherapy appears to have limited efficacy in metastatic TSCTs, including this subtype [4,5,6,26].

Emerging evidence suggests that immune checkpoint inhibitors (ICIs) may offer potential clinical benefit in selected chemotherapy-refractory metastatic cases, with partial responses reported and generally manageable toxicity profiles [27]. However, available data remain extremely limited and require further validation. In the Acosta case series [1], one patient (case no. 11) with IN-TSCT received combined immune checkpoint blockade with ipilimumab and nivolumab after failing multiple lines of chemotherapy—including carboplatin plus paclitaxel and etoposide plus cisplatin—but no clinical response was observed [1].

In the largest available series of metastatic TSCTs, two patients with chemotherapy-refractory disease were treated with ICIs. One patient with Sertoli cell tumor and retroperitoneal lymph node metastasis received sintilimab (200 mg intravenously every 3 weeks) combined with anlotinib (12 mg orally once daily on days 1–14 of a 21-day cycle) for four cycles. Another patient with Leydig cell tumor and metastases to the right lung and left adrenal gland received sintilimab monotherapy (200 mg intravenously every 3 weeks) after failure of first-line chemotherapy. Both patients achieved partial responses, with progression-free survival extending to the last follow-up at 14 and 22 months, respectively. Adverse events were mild (grade 1–2), and no severe immune-related toxicities were reported. These findings suggest that ICIs may provide clinical benefit in selected patients with chemotherapy-resistant disease, particularly given the limited efficacy of platinum-based chemotherapy in metastatic TSCTs [27].

Given the rarity of IN-TSCT, the absence of standardized treatment guidelines, and the tumor’s potential for metastatic progression, management should ideally occur within specialized multidisciplinary teams, including urologic oncologists, medical oncologists, genitourinary pathologists, and molecular tumor boards, to guide therapeutic decision-making and facilitate referral to clinical trials when available. Although responses to ICIs have been rarely reported and appear limited, the manageable safety profile observed in available cases suggests that immunotherapy may be considered in selected patients with metastatic, chemotherapy-refractory disease, with careful monitoring for immune-related adverse events [1,2,27]. Nevertheless, the role of ICIs remains investigational, and their use should be considered on a case-by-case basis in the absence of established effective systemic therapies. Furthermore, predictive biomarkers, expected response rates, and treatment durability remain undefined, particularly given the generally low tumor mutational burden observed in these tumors.

Alterations in *CHEK2* may provide a theoretical rationale for targeted therapeutic strategies that exploit defects in DNA damage response pathways in IN-TSCT. CHEK2 functions as a central mediator of the ATM–CHEK2–p53 signaling cascade activated by DNA double-strand breaks and plays a key role in cell-cycle arrest and homologous recombination repair. Loss-of-function alterations in *CHEK2* may impair homologous recombination proficiency and produce a phenotype analogous to “BRCAness.” In this context, inhibition of poly(ADP-ribose) polymerase (PARP) may induce synthetic lethality by further compromising DNA repair capacity. Although clinical evidence supporting PARP inhibition in IN-TSCT is currently lacking, this mechanistic rationale supports further investigation of PARP inhibitors in tumors harboring *CHEK2* or other homologous recombination repair alterations. However, discussion of PARP inhibition, ALK-directed therapy, or other targeted approaches is currently based primarily on molecular rationale or extrapolation from other tumor types rather than established clinical efficacy in IN-TSCT.

Collectively, because IN-TSCT is an exceptionally rare tumor, therapeutic evidence is largely limited to individual case reports and small case series. Consequently, discussion of systemic treatment strategies must clearly distinguish between approaches directly reported in IN-TSCT and those extrapolated from broader experience with malignant SCSTs or from the biological implications of specific genomic alterations.

## 8. Outcomes and Prognosis

Outcomes for patients with IN-TSCT appear unfavorable in the limited number of reported cases, with relatively high rates of metastasis and recurrence described in the available literature [1,4,5,6]. Survival in advanced disease is often limited, and most patients with metastatic disease have demonstrated poor responses to conventional chemotherapy [4,5,6,9]. Early retroperitoneal lymph node dissection (RPLND) in patients with nodal metastases may improve disease-free survival, although this benefit appears to be largely restricted to patients with nodal-only disease [4,5]. Recent reports describing partial responses to ICIs in chemotherapy-resistant metastatic TSCTs are encouraging; however, their long-term efficacy and safety remain uncertain.

## 9. Knowledge Gaps

There are currently no standardized management guidelines for IN-TSCT, and long-term outcome data remain scarce. Additional research is needed to clarify optimal management strategies, identify prognostic factors, and define the potential role of emerging therapies such as immunotherapy and PARP inhibitors in this rare tumor type [1,2,3,27].

## 10. Conclusions

IN-TSCT represents a distinct, molecularly defined malignant testicular sex cord–stromal tumor characterized by recurrent EWSR1::ATF1 fusion, a unique inflammatory and nested morphology, and potentially aggressive clinical behavior. However, the limited number of reported cases precludes precise estimation of the true metastatic risk and prognostic spectrum of this disease. Accurate recognition requires integration of histologic, immunophenotypic, and molecular findings. Expanded case accrual and multicenter collaborative studies will be essential to refine prognostic stratification and establish evidence-based management strategies.

Although the available literature indicates malignant potential and clinically significant metastatic behavior in several reported cases, these observations should be interpreted with caution given the rarity of the tumor, the small number of reported patients, and the possibility of publication bias toward more aggressive presentations. The rarity of IN-TSCT and the diagnostic and therapeutic challenges associated with this entity underscore the need for continued molecular characterization and investigation of targeted therapeutic approaches.

The presence of *CHEK2* alterations in some cases suggests additional layers of genomic instability that may influence therapeutic strategies. These alterations raise the possibility that tumors harboring homologous recombination repair defects could be susceptible to PARP inhibition, although this hypothesis remains speculative and requires validation in future studies.

## Figures and Tables

**Table 1:** Integrated Molecular and Immunophenotypic Findings in Reported IN-TSCT.

Finding	Acosta et al. 2023 (*n* = 13) [1]	Carillo-Ng et al. 2024 (*n* = 1) [9]	UTh 2025 (*n* = 1) [10]	Wang et al. 2025 (*n* = 1) [8]
Fusion Gene	EWSR1::ATF1 in 10/13	EWSR1::ATF1 detected	EWSR1::ATF1 detected	EWSR1 rearrangement detected (FISH)
Other Fusions	None reported	None reported	None detected	Not reported
TP53 Mutation	Not reported	TP53 p.R248fs*16 (VAF ~70%)	Not detected	Not reported
CHEK2 Mutation	Not reported	Not detected	CHEK2 p.I157T (somatic + germline)	Not reported
MSI Status	Not reported	MSS	MSS	Not reported
Tumor Mutational Burden	Not reported	Low	0.82 muts/Mb (low)	Not reported
ALK Expression (IHC)	Not reported	Not reported	Strong diffuse cytoplasmic	Not reported
PD-L1 Expression	Not reported	Not reported	<1% (negative)	Not reported
HER2 Expression	Not reported	Not reported	Score 0 (negative)	Not reported
CD30 Expression	Positive in 8/10 tested	Diffuse strong	Positive	Diffuse strong positive
β-catenin (nuclear)	Negative	Not reported	Negative	Negative (cytoplasmic only)
SF-1	Positive	Positive	Positive	Negative
Inhibin	Positive	Positive	Positive	Weak positive
EMA	Positive	Positive	Positive	Diffuse positive
Cytokeratin	Variable	Not reported	Positive	Diffuse positive
WT-1	Positive	Not reported	Not reported	Diffuse positive

Cases included in the table are derived from the published literature [1,8,9] unless otherwise specified. The authors’ institutional case, is explicitly labeled as UTh [10].

**Table 2:** Immunophenotypic Overlap Across Gonadal SCSTs.

Marker	IN-TSCT	Granulosa Cell Tumor	Sertoli–Leydig	Sertoli Cell Tumor
SF-1	+	+	+	+
Inhibin	+	+	+	+
WT1	+	+	Variable	Variable
CD30	Frequent +	−	−	−
EMA	+	−	−	Variable
FOXL2	−	+	−	−

**Table 3:** Molecular drivers across testicular and ovarian SCSTs.

Tumor Type	Organ	Defining Molecular Alteration	Pathway	Diagnostic Utility
Adult granulosa cell tumor	Ovary	*FOXL2* C134W	TGF-β/transcriptional regulation	Highly specific
Juvenile granulosa cell tumor	Ovary	*AKT1, GNAS, DICER1*	PI3K/miRNA processing	Subtype defining
Sertoli–Leydig cell tumor	Ovary	*DICER1*	miRNA biogenesis	Germline association
Sertoli cell tumor NOS	Testis	*CTNNB1* mutations	Wnt/β-catenin	Nuclear β-catenin IHC
Large cell calcifying Sertoli cell tumor	Testis	*PRKAR1A*	cAMP signaling	Syndromic association
IN-TSCT	Testis	*EWSR1::ATF1* fusion	CREB transcriptional activation	Defining alteration

## Data Availability

The original contributions presented in this study are included in the article.

## Abbreviations

The following abbreviations are used in this manuscript:

AGCT	Adult granulosa cell tumor
ALK	Anaplastic lymphoma kinase
ATF1	Activating transcription factor 1
AVI	Angiolymphatic invasion
BEP	Bleomycin, etoposide, and platinum
CD	Cluster of differentiation
CREB	cAMP response element-binding protein
CTNNB1	Catenin beta 1 gene
DP	Docetaxel and platinum
EMA	Epithelial membrane antigen
EWSR1	Ewing sarcoma breakpoint region 1 gene
FFPE	Formalin-fixed, paraffin-embedded
FOXL2	Forkhead box L2
FISH	Fluorescence in situ hybridization
GCT	Germ cell tumor
HER2	Human epidermal growth factor receptor 2
H&E	Hematoxylin and eosin
IHC	Immunohistochemistry
ICI	Immune checkpoint inhibitor
IN-TSCT	Inflammatory and nested testicular sex cord tumor
JGCT	Juvenile granulosa cell tumor
LCCSCT	Large cell calcifying Sertoli cell tumor
LN	Lymph node
MSS	Microsatellite stable
NGS	Next-generation sequencing
NOS	Not otherwise specified
OCT3/4	Octamer-binding transcription factor 3/4
PD-1	Programmed cell death protein 1
PD-L1	Programmed death-ligand 1
PI3K	Phosphoinositide 3-kinase
PRKAR1A	Protein kinase A regulatory subunit 1 alpha
RPLND	Retroperitoneal lymph node dissection
SCST	Sex cord–stromal tumor
SF-1	Steroidogenic factor 1
SLCT	Sertoli–Leydig cell tumor
SALL4	Sal-like protein 4
SMAD	Small mothers against decapentaplegic
TGF-β	Transforming growth factor beta
TMB	Tumor mutational burden
TP	Paclitaxel and platinum
TSCT	Testicular sex cord tumor
UTh	University of Thessaly
VAF	Variant allele frequency
WT1	Wilms tumor 1
